# Safety, walking ability, and satisfaction outcomes of the NEURO TRONIC stance-control knee-ankle-foot orthosis (SCKAFO): A comparative evaluation to the E-MAG active SCKAFO

**DOI:** 10.1097/PXR.0000000000000311

**Published:** 2023-11-29

**Authors:** Bart Raijmakers, Merel Anne Brehm, Frans Nollet, Fieke Sophia Koopman

**Affiliations:** 1 Amsterdam UMC location University of Amsterdam, Rehabilitation, Amsterdam, The Netherlands; 2 Amsterdam Movement Sciences, Rehabilitation & Development, Amsterdam, The Netherlands

**Keywords:** orthotics, lower limb, stance control knee-ankle-foot orthoses, stance control orthosis, physical mobility, rehabilitation

## Abstract

**Background:**

Stance control knee-ankle-foot orthoses (SCKAFOs) ensure knee stability by locking during stance while allowing knee flexion during swing. Differences in function of the knee joints and building principles between devices may affect their effectiveness.

**Objective:**

To investigate the preliminary effectiveness of a NEURO TRONIC on safety outcomes, net energy cost (EC), and user experiences in individuals already using an E-MAG Active SCKAFO.

**Study design:**

Prospective uncontrolled intervention study.

**Methods:**

A convenience sample of 10 subjects with flaccid lower extremity muscle weakness, including the quadriceps, due to neuromuscular disorders already using an E-MAG Active SCKAFO were provided with a newly fabricated NEURO TRONIC SCKAFO. Outcomes included knee joint locking failures and unlocking failures (ULFs) (i.e., percentage of steps the knee joint failed to lock/unlock) when walking under challenging conditions on an instrumented treadmill while wearing a safety harness; net EC (J/kg per meter) assessed with a 6-min walk test at comfortable speed; 3D gait kinematics and kinetics; and patient-reported outcomes.

**Results:**

No differences between devices were found for knee joint locking failures (both devices 0%) and ULFs (9.9% for the NEURO TRONIC vs. 13.9% for the E-MAG Active SCKAFO). The mean (standard deviation) net EC with the NEURO TRONIC SCKAFO was 8.2% (from 3.68 [0.81] to 3.38 [0.75] J/kg per meter, *p* = 0.123) lower, although not significantly, compared with that with the E-MAG Active SCKAFO. Significant improvements with the NEURO TRONIC SCKAFO were found for ankle power (*p* = 0.003), perceived walking effort (*p* = 0.014), and reported falls (*p* = 0.034).

**Conclusion:**

Both the NEURO TRONIC SCKAFO and the E-MAG Active SCKAFO were safe in terms of knee joint locking, while ULFs were frequent with both devices. The net EC with the NEURO TRONIC SCKAFO decreased, although not significantly, by 8.2%, likely due to insufficient power. Perceived walking effort was in favor of the NEURO TRONIC SCKAFO.

## Introduction

A variety of neuromuscular disorders (NMDs) cause weakness of the lower extremity muscles including the quadriceps, which may lead to knee instability during standing and walking with increased risk of falling.^1,2^ Knee-ankle-foot orthoses (KAFOs) enhance walking safety by ensuring knee stability and reduce walking energy cost (EC).^3^ While locked KAFOs have been common practice for many decades, nowadays a variety of stance control KAFOs (SCKAFOs) are often applied.^4^ Stance control KAFOs are provided with knee joints that lock only during stance to ensure stability, thereby permitting knee flexion during swing, which results in a more energy efficient gait pattern.^5^

Two frequently applied knee joints for SCKAFOs are the E-MAG Active knee joint (Otto Bock Healthcare, Duderstadt, Germany),^6^ and the NEURO TRONIC knee joint (Fior & Gentz, Lüneburg, Germany).^7^ Both devices are electronically operated. The E-MAG Active knee joint is calibrated to the patient’s step length, measured with a gyroscope built into the SCKAFO, and prelocks in terminal swing at 15 degree knee joint angle, while completely locking upon full knee extension. The NEURO TRONIC knee joint is controlled by motion sensors integrated in the orthosis that detect deceleration of the leg just before initial contact, thereby locking the knee joint at any flexion angle of the knee. Because the NEURO TRONIC knee joint locks at any knee flexion angle, vs. the E-MAG Active knee joint that requires full knee extension to lock, it may be that the NEURO TRONIC knee joint is safer in daily life. For instance, walking on uneven surfaces and handling obstacles or sudden stops may require quick step adjustments with insufficient response time to achieve full knee extension as is required with the E-MAG Active SCKAFO, which, accordingly, could lead lo locking failures (LFs) and related falls. Besides LFs, unlocking failures (ULFs) may also imply a fall risk because prohibiting knee flexion may cause tripping due to insufficient foot clearance in swing. Because both devices unlock at terminal stance or preswing on the condition that an extension moment is acting on the knee joint, we do not expect differences in ULFs between both devices.

In contrast to casting the leg while the patient is lying, the NEURO TRONIC SCKAFO is casted while the patient is standing, using position sensors to assure that the orthosis aligns with the leg position during gait. This way of alignment could improve the gait pattern^8^ and consequently lower walking EC, which is important because walking-related fatigue is a common reported problem in individuals with NMD.^9^

Because a NEURO TRONIC SCKAFO differs from an E-MAG Active SCKAFO in terms of locking mechanism while walking and casting technique, in this explorative study, we investigated the preliminary effectiveness of a NEURO TRONIC SCKAFO on: (1) knee joint functioning expressed as LF and ULFs when walking under challenging conditions, (2) net EC at comfortable speed, (3) gait kinematics and kinetics, and (4) perceived walking ability, satisfaction, and reported number of falls in subjects with lower extremity muscle weakness due to NMD who were previously provided with an E-MAG Active SCKAFO.

## Methods

### Study design

This prospective uncontrolled intervention study was performed at the Department of Rehabilitation Medicine at the Amsterdam University Medical Centre, Academic Medical Center, Amsterdam, The Netherlands. The study protocol was approved by the AMC Medical Ethics Committee and registered at www.trialregister.nl (registration ID: NL6892). Reporting was according to the Strengthening the Reporting of Observational Studies in Epidemiology (STROBE) guidelines.^10^

### Research participants

The sample size for this study was based on feasibility in terms of availability of eligible subjects and their expected willingness to participate. Between March 2018 and February 2020, we enrolled 10 subjects with flaccid lower extremity muscle weakness, including the quadriceps, due to a variety of NMDs. Subjects were recruited from the Department of Rehabilitation Medicine at the Academic Medical Center and the orthopedic company involved in the study (OIM Orthopedie, Noordwijkerhout, The Netherlands). Inclusion criteria were as follows: (1) age between 18 and 80 years; (2) currently using an E-MAG Active SCKAFO at least when walking outside for more than 3 months; and (3) able to walk 6 mins continuously at comfortable speed. Exclusion criteria were as follows: (1) no SCKAFO indication upon examination; (2) >10 degrees uncorrectable knee varus or valgus deformity; and (3) >10 degrees knee flexion contracture. All subjects provided written informed consent before inclusion.

### Protocol

**Figure 1.**
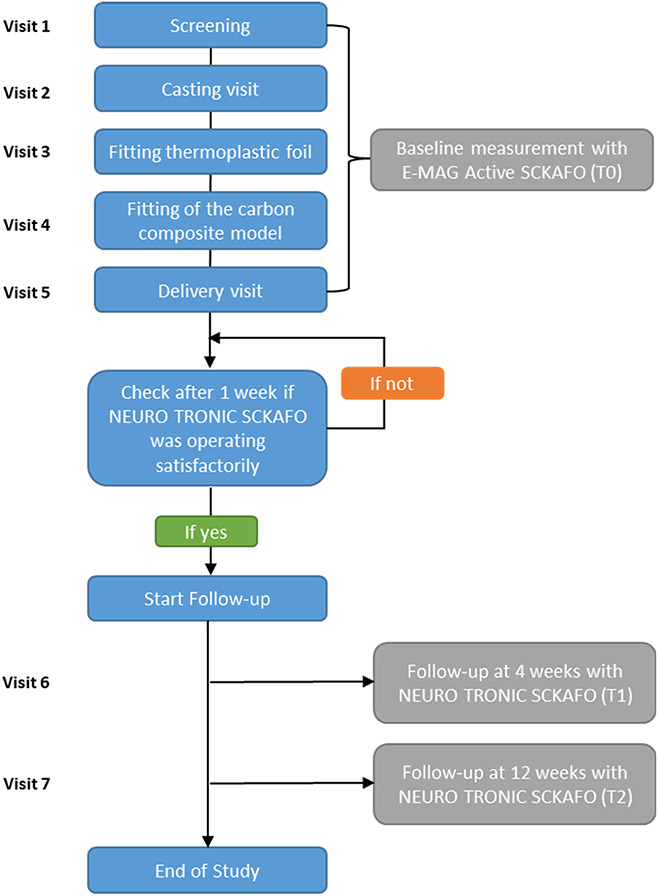
Study overview.

Figure 1 shows the study protocol overview. Subjects visited the outpatient clinic for consultations with a rehabilitation physician, certified prosthetist/orthotist (CPO), and physical therapist for a minimum of 7 times. The first 5 visits involved the screening, casting, fitting, and delivery of the NEURO TRONIC SCKAFO and the baseline measurements with the E-MAG Active SCKAFO. If necessary, additional visits for further adjustments of the NEURO TRONIC SCKAFO were scheduled. When the NEURO TRONIC SCKAFO was delivered satisfactorily, the follow-up visits were planned.

### Intervention

#### Baseline orthosis: E-MAG active SCKAFO

The E-MAG Active SCKAFOs had been provided in usual care in different centers in the Netherlands by a CPO on the prescription of a rehabilitation physician. All E-MAG Active SCKAFOs were made based on a plaster cast with subjects lying, and the joint angles on the cast were determined by hand (conventional model technique). All E-MAG Active SCKAFOs were made of carbon composite and equipped with hinged dorsiflexion stop ankle joints or solid ankle joints (Appendix, http://links.lww.com/POI/A208).

#### Experimental orthosis: NEURO TRONIC SCKAFO

The NEURO TRONIC SCKAFOs were composed of carbon composite (Appendix, http://links.lww.com/POI/A208). The orthosis configurator of Fior & Gentz^11^ was used to decide on the construction of the SCKAFO, and the type of ankle joint (options: hinged ankle joint with dorsiflexion stop, spring-hinged ankle joint, or solid ankle joint). Before making the plaster cast, a dynamic static position was determined, mimicking the alignment of the limbs and center of mass in a gait corresponding posture, with subjects standing and partially loading the affected leg, referred to as physiological modeling technique.^8^ 3D position sensors attached to the subjects’ thigh, lower leg, and foot measured the segmental orientation of this dynamic position, which were transferred to the plaster cast^12^ (Appendix, http://links.lww.com/POI/A208).

### Outcome measures

For familiarization and to account for known learning effects,^13^ the primary outcome “knee joint functioning while walking under challenging conditions” was measured twice for the E-MAG Active KAFO at baseline, using the second measurement for analysis. All other outcomes were measured once for the E-MAG Active SCKAFO at baseline (T0) and for the NEURO TRONIC SCKAFO at 4 (T1) and 12 weeks (T2) follow-up. All outcomes were collected, postprocessed, and entered into a Castor database by 1 trained researcher (B.R.).

### Demographic and clinical characteristics

At baseline, sociodemographic and clinical characteristics were assessed. Furthermore, properties of both SCKAFO types were inventoried.

### Primary outcomes

#### Knee joint functioning

Knee joint functioning while walking under challenging conditions was assessed on an interactive treadmill: the C-Mill (Motek, Amsterdam, The Netherlands), which can accurately project stepping targets or obstacles onto the belt while walking.^14^ The C-Mill has previously been used in successfully assessing walking adaptability in polio survivors, including SCKAFO users.^14^

**Figure 2.**
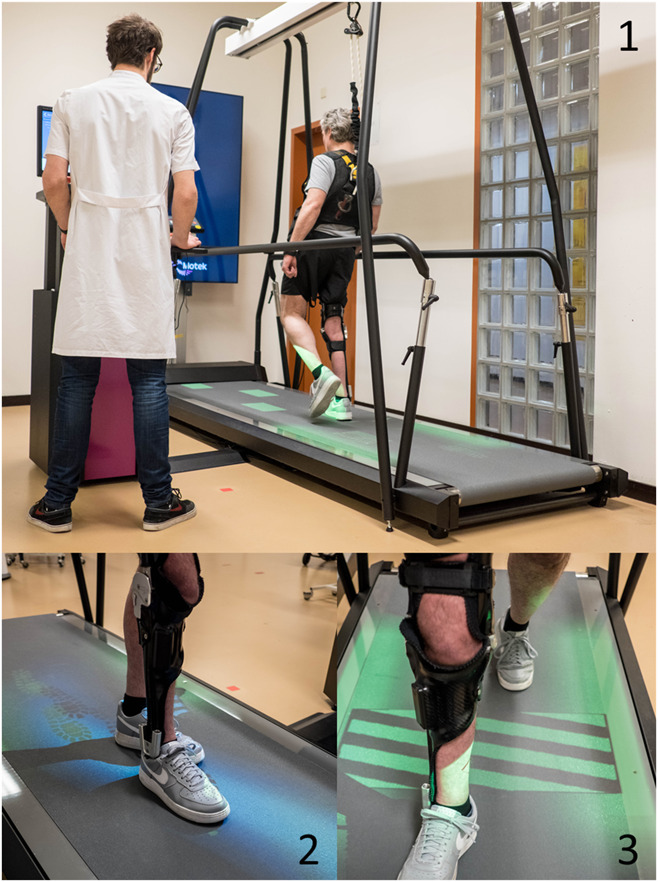
C-Mill interactive treadmill with safety harness (upper picture) and visual projections onto the belt. (1) Target stepping: rectangular stepping targets were projected onto the belt for left and right steps for a duration of 2 mins. Targets were presented with 20% variation in step length and width as derived from 15-s steady-state walking before target presentation. Subjects were instructed to place their feet in the center of the stepping targets. (2) Obstacle avoidance: obstacles were projected on the belt in an anticipatory condition and then in a reactive condition in which the obstacles appeared 4 steps and 1 step ahead of the subject, respectively. Twelve obstacles (6 in the anticipatory condition and 6 in the reactive condition) were projected, and subjects were instructed to avoid the obstacles with both the swing and stance leg. (3) Slalom walking: a beam, 20 cm wide, was projected with slalom bends onto the belt. Subjects were instructed to place their feet within the projected beam and follow the projected path for a period of 2 mins.

After a familiarization protocol, comfortable walking speed was determined for all subjects as previously reported.^15^ This speed was used during all tests, which included (1) target stepping, (2) obstacle avoidance, and (3) slalom walking (Figure 2). Subjects wore a safety harness to prevent falling due to possible knee joint LF or ULFs and rested at least 2 mins between tests until recovered. When the subject was reliant on a walking aid, handrail use was allowed.

Continuous video recordings in the sagittal and frontal planes were made to evaluate knee joint functioning. Knee joint functioning was determined by scoring the total number of knee joint LFs during all challenging conditions on the C-Mill divided by total step count (in %). An LF was defined as *knee flexion movement in the SCKAFO leg during the stance phase that led to a (near) fall*. Besides LFs, ULFs were determined and calculated as the total number of ULFs during all challenging conditions on the C-Mill divided by total step count (in %). A ULF was defined *as absence of knee flexion during the swing phase*. The first 10 steps were excluded from the analysis of LF/ULF due to the treadmill speeding up to preferred comfortable speed. Locking failure and ULF were scored by 2 investigators (BR, FSK) independently and checked for consistency. In case of inconsistencies, events were discussed with the research group for a unanimous agreement.

#### Net EC

The net EC was assessed during a 6-minute walk test (6MWT). First, an 8-min rest test was performed while sitting on a chair to determine energy consumption (J/kg per seconds) during rest. Subjects were instructed not to eat or drink anything at least 90 mins before the test. Subsequently, the 6MWT was performed at a self-selected, comfortable walking speed on an indoor oval track (38 m). Subjects could use their customary walking aid(s), if needed. Oxygen uptake (VO_2_) and the respiratory exchange ratio (RER) were measured breath-by-breath with a portable gas analysis system (Cosmed K5a, Rome, Italy). Mean VO_2_, RER, and walking speed values were obtained from a visually determined steady-state period of at least 60 s from the last 3 mins of the rest test and the 6MWT. With these values, the average steady-state net energy consumption was calculated, using a custom-written Matlab script (V.2019; MathWorks, Natick, Massachusetts), as: ([4.940 × RER + 16.040] × VO_2_^16^), expressed in J/kg per minute. Finally, net EC was calculated by dividing net energy consumption by steady-state walking speed, expressed in J/kg per meter. Walking EC can be reliably measured in subjects with NMDs.^17,18^

### Secondary outcomes

#### Gait kinematics and kinetics

3D gait analyses were performed at comfortable walking speed along a 12-m long walkway with a 100-Hz 12-camera 3D motion capture system (VICON MX 1.3) and 2 1000-Hz force plates in series (OR 6–7; AMTI, Watertown, Marssachusetts). Reflective 14-mm passive body markers were placed on anatomical landmarks to fit the Plug-in-Gait model used within the VICON/Nexus software package. A minimum of 3 valid gait trials was collected. A trial was considered valid if the subject stepped on a force plate with 1 foot, and all markers are visible from heel strike until ipsilateral heel strike. For each trial, joint angles, net joint moments, and joint powers around the ankle, knee, and hip were time normalized to the gait cycle (0%–100%). Subsequently, the 3 trials were averaged, and the following 3D gait parameters of the subjects’ leg in the sagittal and frontal planes were calculated for each phase of the gait cycle: maximum and mean ankle angle/moment/power (sagittal plane); and maximum and mean knee and hip angles/moments (sagittal and frontal planes), and spatiotemporal parameters. Note that the frontal knee angle and moment were analyzed to evaluate the change in frontal alignment (correction of varus and valgus deformations) between both KAFOs. Therefore, these outcomes were transformed into absolute values to correct for the leveling effect of varus (negative values) and valgus (positive values) deformations, which were both present in most of the included subjects, on mean outcomes.

#### Perceived effectiveness, satisfaction, and reported falls

An in-house questionnaire was used to assess perceived effectiveness and satisfaction on standing and walking ability with the SCKAFO and reported falls. Perceived effectiveness was measured for 16 items related to standing/walking with the SCKAFOs, all scored on a 7-point Likert scale (very badly [1] to very well [7]). Satisfaction was measured for the same items related to standing/walking with the SCKAFOs (except for “number of falls”) and 2 items on looks and fitting, all scored on a 5-point Likert scale (totally not satisfied [1] to very satisfied [5]). For each item, perceived effectiveness and satisfaction were expressed as the score for the NEURO TRONIC SCKAFO minus the score for the E-MAG Active SCKAFO. In addition, an average overall score was calculated by taking the mean difference of all items. Satisfaction with the KAFO was also measured with the Dutch version of the Quebec User Evaluation of Satisfaction with Assistive Technology (D-QUEST) questionnaire,^19^ which has good psychometric properties.^20^ The reported number of falls was assessed as number of falls per day/week/month/year and converted to number of falls per year.

### Statistical analysis

SPSS version 28 (IBM SPSS, Chicago, Illinois) was used for all statistical analyses, and significance levels were set at *p* < 0.05. Data were checked for normality with skewness and kurtosis calculations, and QQ plots and Shapiro-Wilk tests were performed for normality. Accordingly, summary statistics were calculated for participant characteristics and for all outcomes. Differences between the E-MAG Active SCKAFO at baseline (T0) and the NEURO TRONIC SCKAFO at 3-month follow up (T2) were analyzed with paired samples *t* tests for continuous outcomes (LF, ULF, walking speed, net EC, and gait parameters) and with Wilcoxon signed rank tests for ordinal data (perceived effectiveness, satisfaction, and reported falls). We used the T2 time point and not T1 to allow subjects enough time to optimize walking with the NEURO TRONIC SCKAFO.^21^ Last observation carried forward was used as a method of imputing missing data, in case T2 data were missing.

## Results

### Participant characteristics

One subject withdrew consent after inclusion due to the Covid-19 circumstances; therefore, analyses were performed on 9 subjects (8 polio survivors and 1 subject with postradiation lumbosacral plexopathy; mean [standard deviation (SD)] age: 60.3 [10.5] years). Seven subjects used their E-MAG Active SCKAFO at least 4 d a week (Table 1), which was identical at follow-up for the NEURO TRONIC SCKAFO. Characteristics of both SCKAFOs are summarized in Table 1.

**Table 1.**
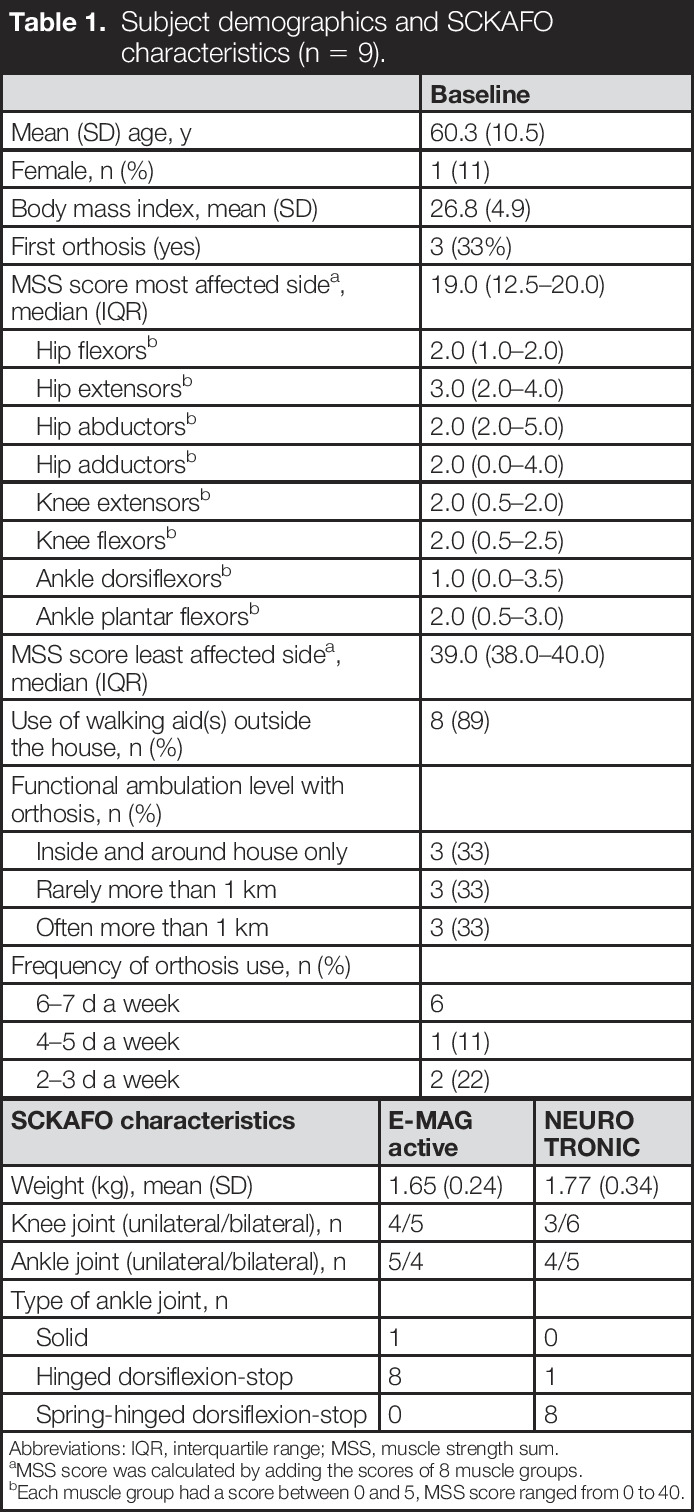
Subject demographics and SCKAFO characteristics (n = 9).

	Baseline
Mean (SD) age, y	60.3 (10.5)
Female, n (%)	1 (11)
Body mass index, mean (SD)	26.8 (4.9)
First orthosis (yes)	3 (33%)
MSS score most affected side^a^, median (IQR)	19.0 (12.5–20.0)
Hip flexors^b^	2.0 (1.0–2.0)
Hip extensors^b^	3.0 (2.0–4.0)
Hip abductors^b^	2.0 (2.0–5.0)
Hip adductors^b^	2.0 (0.0–4.0)
Knee extensors^b^	2.0 (0.5–2.0)
Knee flexors^b^	2.0 (0.5–2.5)
Ankle dorsiflexors^b^	1.0 (0.0–3.5)
Ankle plantar flexors^b^	2.0 (0.5–3.0)
MSS score least affected side^a^, median (IQR)	39.0 (38.0–40.0)
Use of walking aid(s) outside the house, n (%)	8 (89)
Functional ambulation level with orthosis, n (%)	
Inside and around house only	3 (33)
Rarely more than 1 km	3 (33)
Often more than 1 km	3 (33)
Frequency of orthosis use, n (%)	
6–7 d a week	6
4–5 d a week	1 (11)
2–3 d a week	2 (22)

SCKAFO characteristics	E-MAG active	NEURO TRONIC
Weight (kg), mean (SD)	1.65 (0.24)	1.77 (0.34)
Knee joint (unilateral/bilateral), n	4/5	3/6
Ankle joint (unilateral/bilateral), n	5/4	4/5
Type of ankle joint, n		
Solid	1	0
Hinged dorsiflexion-stop	8	1
Spring-hinged dorsiflexion-stop	0	8

Abbreviations: IQR, interquartile range; MSS, muscle strength sum.

a MSS score was calculated by adding the scores of 8 muscle groups.

b Each muscle group had a score between 0 and 5, MSS score ranged from 0 to 40.

### Knee joint functioning

During the tests on the C-Mill, LF did not occur with the NEURO TRONIC SCKAFO and not with the E-MAG Active SCKAFO. The % ULF was 9.3% for the NEURO TRONIC SCKAFO vs. 13.9% for the E-MAG Active SCKAFO (*p* = 0.406) (Table 2). The mean (SD) comfortable treadmill speed increased significantly with 0.11 m/s (+19%, *p* = 0.029), from 0.59 (0.17) m/s for the E-MAG Active SCKAFO to 0.70 (0.15) m/s for the NEURO TRONIC SCKAFO.

**Table 2.**
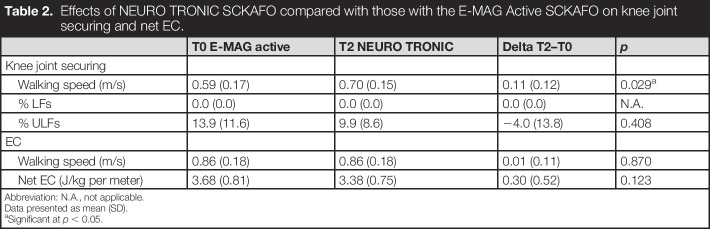
Effects of NEURO TRONIC SCKAFO compared with those with the E-MAG Active SCKAFO on knee joint securing and net EC.

	T0 E-MAG active	T2 NEURO TRONIC	Delta T2–T0	*p*
Knee joint securing				
Walking speed (m/s)	0.59 (0.17)	0.70 (0.15)	0.11 (0.12)	0.029^a^
% LFs	0.0 (0.0)	0.0 (0.0)	0.0 (0.0)	N.A.
% ULFs	13.9 (11.6)	9.9 (8.6)	−4.0 (13.8)	0.408
EC				
Walking speed (m/s)	0.86 (0.18)	0.86 (0.18)	0.01 (0.11)	0.870
Net EC (J/kg per meter)	3.68 (0.81)	3.38 (0.75)	0.30 (0.52)	0.123

Abbreviation: N.A., not applicable.

Data presented as mean (SD).

a Significant at *p* < 0.05.

### Net EC

The mean (SD) comfortable walking speed during the 6MWT was similar between the E-MAG Active SCKAFO and NEURO TRONIC SCKAFO (both: 0.86 [0.18] m/s, *p* = 0.870). The net EC was −0.30 J/kg per meter (−8.2%) lower with the NEURO TRONIC SCKAFO (3.38 [0.75] J/kg per meter) compared that with the E-MAG Active SCKAFO (3.68 [0.81] J/kg per meter), which difference was not significant (*p* = 0.123). Six subjects reduced in net EC beyond the standard error of measurement of 0.173 J/kg per meter,^17^ ranging from −0.28 to −1.34 J/kg per meter, while 1 subject showed no difference (+0.06 J/kg per meter) and 2 subjects showed an increment in net EC of +0.20 and +0.48 J/kg per meter.

### Spatiotemporal parameters and gait kinematics and kinetics

**Figure 3.**
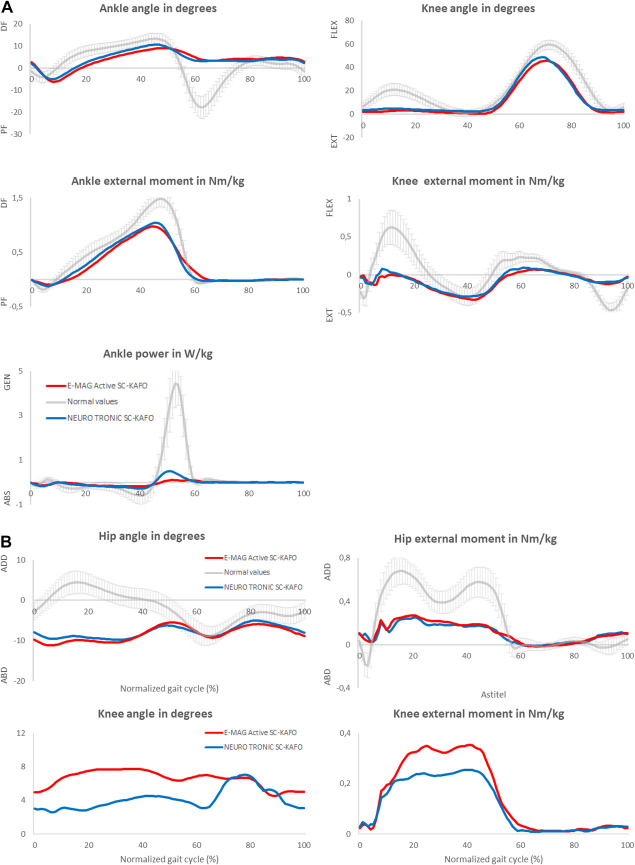
Gait biomechanics. Graphs are shown as percentage of the normalized gait cycle (0%–100%). Normal values are average data from 28 healthy controls measured in our own human performance laboratory. (a) (sagittal plane): DF: dorsiflexion, PF: plantar flexion, EXT: extension, FLEX: flexion, W: Watt, Nm: Newton meter, kg: kilogram, Gen: generation, Abs: absorption. (b) (frontal plane): ABD: abduction, ADD: adduction. Nm: Newton meter, kg: kilogram, frontal knee angle and frontal knee external moment are shown as absolute mean values.

Analyses of 3D gait kinematics and kinetics were performed on 8 subjects because 1 subject was unable to walk without a walking aid at the follow-up measurement. No differences were observed between SCKAFOs in any of the spatiotemporal parameters (*p* ≥ 0.148) (Table 3). Sagittal and frontal ankle and knee and hip kinematics and kinetics did not differ between both types (*p* ≥ 0.084), except for 2 gait parameters. Maximal ankle power in terminal stance increased significantly with the NEURO TRONIC (0.77 [0.32] W/kg) compared with the E-MAG Active (0.35 [0.12] W/kg, *p* = 0.003) (Figure 3(a)). In addition, the frontal knee moment during single stance decreased significantly with the NEURO TRONIC (0.23 [0.15] Nm/kg) compared with the E-MAG Active (0.31 [0.16] Nm/kg, *p* = 0.008) (Figure 3(b)).

**Table 3.**
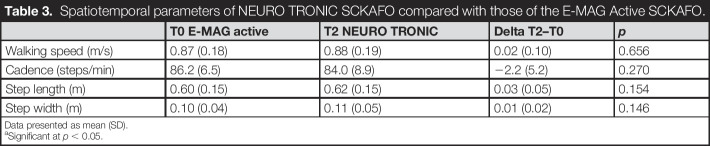
Spatiotemporal parameters of NEURO TRONIC SCKAFO compared with those of the E-MAG Active SCKAFO.

	T0 E-MAG active	T2 NEURO TRONIC	Delta T2–T0	*p*
Walking speed (m/s)	0.87 (0.18)	0.88 (0.19)	0.02 (0.10)	0.656
Cadence (steps/min)	86.2 (6.5)	84.0 (8.9)	−2.2 (5.2)	0.270
Step length (m)	0.60 (0.15)	0.62 (0.15)	0.03 (0.05)	0.154
Step width (m)	0.10 (0.04)	0.11 (0.05)	0.01 (0.02)	0.146

Data presented as mean (SD).

a Significant at *p* < 0.05.

### Perceived effectiveness, satisfaction, and reported falls

The median (interquartile range) perceived effectiveness on walking effort improved significantly with the NEURO TRONIC SCKAFO (4.0 [3.0–6.0]) compared with that with the E-MAG Active SCKAFO (3.0 [2.0–5.0], *p* = 0.014]). All other items and overall perceived effectiveness did not differ between SCKAFO types (Table 4).

**Table 4.**
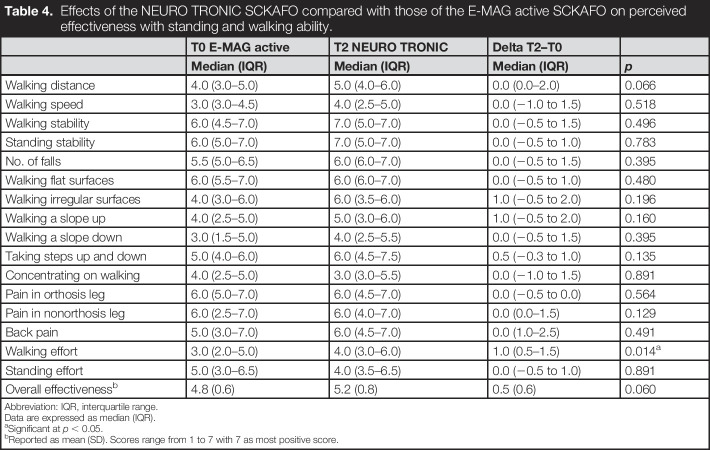
Effects of the NEURO TRONIC SCKAFO compared with those of the E-MAG active SCKAFO on perceived effectiveness with standing and walking ability.

	T0 E-MAG active	T2 NEURO TRONIC	Delta T2–T0	
	Median (IQR)	Median (IQR)	Median (IQR)	*p*
Walking distance	4.0 (3.0–5.0)	5.0 (4.0–6.0)	0.0 (0.0–2.0)	0.066
Walking speed	3.0 (3.0–4.5)	4.0 (2.5–5.0)	0.0 (−1.0 to 1.5)	0.518
Walking stability	6.0 (4.5–7.0)	7.0 (5.0–7.0)	0.0 (−0.5 to 1.5)	0.496
Standing stability	6.0 (5.0–7.0)	7.0 (5.0–7.0)	0.0 (−0.5 to 1.0)	0.783
No. of falls	5.5 (5.0–6.5)	6.0 (6.0–7.0)	0.0 (−0.5 to 1.5)	0.395
Walking flat surfaces	6.0 (5.5–7.0)	6.0 (6.0–7.0)	0.0 (−0.5 to 1.0)	0.480
Walking irregular surfaces	4.0 (3.0–6.0)	6.0 (3.5–6.0)	1.0 (−0.5 to 2.0)	0.196
Walking a slope up	4.0 (2.5–5.0)	5.0 (3.0–6.0)	1.0 (−0.5 to 2.0)	0.160
Walking a slope down	3.0 (1.5–5.0)	4.0 (2.5–5.5)	0.0 (−0.5 to 1.5)	0.395
Taking steps up and down	5.0 (4.0–6.0)	6.0 (4.5–7.5)	0.5 (−0.3 to 1.0)	0.135
Concentrating on walking	4.0 (2.5–5.0)	3.0 (3.0–5.5)	0.0 (−1.0 to 1.5)	0.891
Pain in orthosis leg	6.0 (5.0–7.0)	6.0 (4.5–7.0)	0.0 (−0.5 to 0.0)	0.564
Pain in nonorthosis leg	6.0 (2.5–7.0)	6.0 (4.0–7.0)	0.0 (0.0–1.5)	0.129
Back pain	5.0 (3.0–7.0)	6.0 (4.5–7.0)	0.0 (1.0–2.5)	0.491
Walking effort	3.0 (2.0–5.0)	4.0 (3.0–6.0)	1.0 (0.5–1.5)	0.014^a^
Standing effort	5.0 (3.0–6.5)	4.0 (3.5–6.5)	0.0 (−0.5 to 1.0)	0.891
Overall effectiveness^b^	4.8 (0.6)	5.2 (0.8)	0.5 (0.6)	0.060

Abbreviation: IQR, interquartile range.

Data are expressed as median (IQR).

a Significant at *p* < 0.05.

b Reported as mean (SD). Scores range from 1 to 7 with 7 as most positive score.

Satisfaction, both per item and overall, did not differ between SCKAFO types. In addition, there was no difference in the mean (SD) score of satisfaction with orthosis-related aspects (both SCKAFOs: 4.0 [0.5], *p* = 0.856) and service-related aspects (E-MAG Active SCKAFO: 4.3 [0.6] vs. NEURO TRONIC SCKAFO: 4.5 [0.5], *p* = 0.354) measured with the D-QUEST.

The median (interquartile range) reported number of falls was significantly lower for the NEURO TRONIC SCKAFO (0.0 [0.0–3.5]) than for the E-MAG Active SCKAFO (1.0 [0.0–10.0], *p* = 0.034).

## Discussion

Our study in adults with knee instability due to lower extremity muscle weakness showed that a NEURO TRONIC SCKAFO and an E-MAG Active SCKAFO are equally safe in terms of knee joint locking and unlocking during adaptive walking on an instrumented treadmill. The net EC with the NEURO TRONIC SCKAFO was 8.2% lower compared with that with E-MAG Active SCKAFO, although not significantly. A lower perceived walking effort in daily life was reported for the NEURO TRONIC SCKAFO compared with that for the E-MAG Active SCKAFO, while overall perceived effectiveness and satisfaction were comparable between both SCKAFO types.

Because this was the first study to evaluate walking safety for SCKAFOs in terms of knee joint functioning, our results cannot be compared with those of previous studies. It is noticeable that no LFs occurred during provocative walking tests on the treadmill because falling in daily life with SCKAFOs has been reported in others studies^21,22^ and in our own study. Possibly because the walk tests on the C-Mill treadmill were not provocative enough. In addition, the inclusion of subjects who were already used to walking with a SCKAFO could have been of influence because they were perhaps sufficiently skilled to adequately use their SCKAFO during the challenging walk tests presented on the treadmill. This inability to provoke stumbling or falling when performing challenging walk tasks has been shown before in studies on individuals accustomed to different types of prosthetic knee joints.^23,24^

Besides LFs, we also studied the number of ULFs. Although we did not expect differences in ULFs between both SCKAFOs, the number of ULFs was relatively high for both SCKAFO types and with large interindividual differences. Unlocking failures are associated with a higher cognitive load during walking^25,26^ and can provoke unsafe events because foot clearance is hampered by swinging forward a stiff leg. To alleviate these events and provide a safer, smooth gait, more insight is needed into the mechanism behind ULFs. Noteworthy is the higher treadmill speed during the challenging walk tests with the NEURO TRONIC SCKAFO compared with that with the E-MAG Active SCKAFO, which may suggest that subjects felt more confident when walking with the NEURO TRONIC SCKAFO, although a learning effect of walking on the treadmill cannot be ruled out,^27,28^ because no differences were found in overground walking speed.

Our study showed conflicting results regarding falling. Reported falls were significantly lower with the NEURO TRONIC SCKAFO compared with those with the E-MAG Active SCKAFO, while no difference of perceived effectiveness on number of falls (Table 4) between both SCKAFOs was found. Therefore, definite conclusions on safety of walking with both SCKAFOs in daily life could not be drawn. Because the circumstances of reported falls were not inventoried, factors related to differences in walking safety in daily life between the SCKAFO types cannot be compared. Therefore, better insight on stance control knee joint functioning and related falls in daily life is needed. This would require more ecologically valid outcome measures to mirror the challenges of daily life walking and the (future) possibility to read out data from stance control knee joints. Given the striking difference in occurrence of LF and ULFs during the treadmill tests, it may well be that falling in daily life is related with ULFs rather than with LFs.

The net EC with the NEURO TRONIC SCKAFO reduced by 8.2% compared with that with the E-MAG Active SCKAFO. This reduction was not significant, likely due to the small sample size and the large heterogeneity of the data, expressed by the large SD change. Yet the reduction in EC found in our study is comparable with a previous study that reported a similar but significant reduction of 8% in net EC in a larger sample of polio survivors (n = 20) who walked with locked KAFOs^18^ due to optimization of materials (i.e., carbon composite), fitting, and biomechanical effectiveness of the applied ankle joints. In line with our finding that walking with a NEURO TRONIC SCKAFO seems more energy effective than walking with an E-MAG Active SCKAFO, experienced walking effort improved significantly in favor of the NEURO TRONIC SCKAFO, indicating that daily life walking was perceived energetically less demanding.

In trying to explain the energetic benefit of the NEURO TRONIC SCKAFO compared with that of the E-MAG-Active SCKAFO, we argue that the reduction in net EC and in perceived walking effort might be explained by 2 possible mechanisms found in our participants. First, the 3D gait analysis also showed an increased ankle power with the NEURO TRONIC SCKAFO compared with that with the E-MAG Active SCKAFO, which was present in all subjects. It is likely that this increase in ankle power may have, partially, resulted from the spring-hinged ankle joints used in the NEURO TRONIC SCKAFOs (applied in 8 of 9 participants) because the properties of this ankle joint have been demonstrated to increase ankle power^29,30^ compared with solid or hinged dorsiflexion restricting ankle joints, as were used in the E-MAG Active SCKAFOs. Second, we found a reduction in the frontal knee moment during single stance with the NEURO TRONIC SCKAFO. Likely, this assumed positive effect was attributed to the more straight upbuild of the NEURO TRONIC SCKAFO due to the physiological casting technique, determined from the, nonsignificant, reduced frontal knee joint angle during single stance (Figure 3). Unfortunately, due to the small sample size, we could not perform a more in-depth correlation analysis on change in frontal knee moment and change in net EC, and therefore, the results of this study are insufficient to draw conclusions on the role of the physiological casting technique and reduction of frontal knee moment on the reduction in net EC. A study with a larger sample size should be performed to explore the role of casting techniques on (SC)KAFO alignment, gait pattern, and EC of walking.

Besides the aforementioned improvement in perceived walking effort, we found no differences for other aspects of perceived effectiveness or satisfaction. The lack of differences between the 2 SCKAFO types may signify that our subjects were already walking satisfactorily with their E-MAG Active SCKAFO, as indicated by the median satisfaction score of 4.0 on the D-QUEST at baseline, which was indeed already high.^19^

## Limitations

Some limitations should be considered. Our study had a small sample size, which affected the statistical power, making it harder to draw firm conclusions on the investigated devices. Studies on (SC)KAFOs would benefit from larger sample sizes when comparing KAFO properties, such as joints, materials, and building principles. In this way, the impact of these individual aspects on functioning with the SCKAFO can be better elucidated. Because KAFO research is very elaborate in nature, this would require costly, large-scaled multicenter studies. In addition, our inclusion of satisfied E-MAG Active SCKAFO users could have led to selection bias. It is possible that less satisfied E-MAG Active SCKAFO users could have experienced a more positive effect on outcomes when provided with a NEURO TRONIC SCKAFO. Finally, the SCKAFOs used in this study are expensive devices in terms of used materials and fabrication costs, which will limit their applicability in certain regions worldwide.

## Conclusion

Our small-sized study in adults with knee instability due to lower extremity muscle weakness indicates that in terms of knee joint safety, evaluated in a laboratory setting, the E-MAG Active SCKAFO and the NEURO TRONIC SCKAFO have comparable outcomes. The net EC reduced by 8.2%, but not significantly, with the NEURO TRONIC SCKAFO compared with that with the E-MAG Active SCKAFO, and perceived walking effort was in favor of the NEURO TRONIC SCKAFO. Larger controlled randomized studies are warranted to compare differences in effectiveness between devices.

## Funding

The authors disclosed receipt of the following financial support for the research, authorship, and/or publication of this article: This work was financially supported by Fior & Gentz, Lüneburg, Germany, the manufacturer of the NEURO TRONIC SC knee joint and OIM Orthopedie, Assen, The Netherlands. In addition, we received in-kind support because Fior & Gentz provided the NEURO TRONIC SC knee joints used in the study and OIM Orthopedie manufactured the SCKAFOs with the integrated NEURO TRONIC SC knee joint. Fior & Gentz and OIM Orthopedie had no role in the collection, analysis, and interpretation of the data. Both parties have reviewed the manuscript to check whether it contained proprietary information that had to be held confidential. If applicable, all parties discussed how to adapt the proposed publication. Any deletions or changes requested by Fior & Gentz and OIM Orthopedie have not involved the results of the study.

## Supplemental material

Supplemental material for this article is available in this article. Direct URL citation appears in the text and is provided in the HTML and PDF versions of this article on the journal’s Web site (www.POIjournal.org).
